# Frequency and Genotype of Hepatitis D Virus Infection in Patients Infected with HIV and Those Undergoing Hemodialysis

**DOI:** 10.5812/hepatmon.7481

**Published:** 2013-05-11

**Authors:** Mohammad Reza Aghasadeghi, Minoo Mohraz, Golnaz Bahramali, Arezoo Aghakhani, Mohammad Banifazl, Maryam Foroughi, Farrokhlagha Ahmadi, Ali Eslamifar, Seyed Mehdi Sadat, Amitis Ramezani

**Affiliations:** 1 Department of Hepatitis and AIDS, Pasteur Institute, Tehran, IR Iran; 2 Iranian Research Center for HIV/AIDS, Tehran, IR Iran; 3 Department of Clinical Research, Pasteur Institute, Tehran, IR Iran; 4 Iranian Society for Support of Patients With Infectious Diseases, Tehran, IR Iran; 5 Nephrology Research Center, Tehran University of Medical Sciences, Tehran, IR Iran

**Keywords:** Hepatitis Delta Virus, HIV, Hemodialysis, Genotype

## Abstract

**Background:**

Hepatitis D virus (HDV) is a defective virus dependent on hepatitis B virus (HBV) for its replication. Due to HDV transmission routes, patients undergoing hemodialysis and those with HIV infection are at risk of acquiring HDV.

**Objectives:**

This study was aimed to determine the frequency and genotype of HDV infection among patients with HIV infection and those undergoing hemodialysis.

**Patients and Methods:**

720 cases including 120 patients undergoing hemodialysis, and 600 patients with HIV infection were studied. All cases with positive results for HBsAg were evaluated for the presence of anti-HDV antibodies. Samples with Anti-HDV positive results were subjected to nested PCR for HDV-RNA confirmation, and sequenced for HDV genotype determination.

**Results:**

HBsAg was found in 9 (7.5%) of 120 patients undergoing hemodialysis, and 9 (1.5%) of 600 patients with HIV infection. 3 (33.3%) of patients undergoing hemodialysis with positive results for HBsAg, and 5 (55.5%) of cases with HIV infection and positive results for HBsAg, had positive findings for anti-HDV which were then subjected to nested PCR. The amplification results confirmed that in 3 (37.5%) samples HDV-RNA was detected. Overall 2.5% of patients undergoing hemodialysis, and 0.8% of cases infected with HIV had positive results for anti-HDV and 1.7% and 0.2% of cases undergoing hemodialysis and patients infected with HIV had positive findings for HDV-RNA respectively. All of the HDV isolates were clustered in clade 1.

**Conclusions:**

The survey showed that overall HDV frequency was not high in our high risk cases. Therefore, practitioners and health care managers should become aware of the risk of dual infection with HBV and HDV especially in high risk patients.

## 1. Background

Hepatitis D virus (HDV) is a defective satellite virus which its infectivity is dependent on hepatitis B virus (HBV) and affects nearly 20 million people worldwide (1). HDV infection leads to more severe liver disease than HBV monoinfection with accelerated fibrosis progression, earlier hepatic decompensation and an increased risk for the development of hepatocellular carcinoma (2). High prevalence areas for HDV infection include Italy, parts of Eastern Europe, the Amazon basin, Venezuela, Columbia, some Pacific Islands, Pakistan and Western Asia and infection apparently endemic in the Middle East (3-8). Studies from different areas of Iran showed varied prevalence rates of HDV infection from 2.4% to 5.8% in HBV carriers (9, 10). The prevalence of HDV in various high risk groups in Iran was studied in few surveys. (11-14). In Tehran, HDV prevalence was found to be 5.7% in patients with chronic HBV in 2004 (15). Three distinct genotypes and two subtypes are described for HDV. Genotype I is distributed worldwide, but other genotypes appear to be more restricted geographically. Genotype _Ι_ is spread in the United States, the Middle East, and Europe (7, 16). Genotype _ΙΙ_ is predominant in the Far East (17) and Genotype _ΙΙΙ_ is predominantly detected in South America (3). The predominant genotype of HDV reported from Iran is Genotype _Ι_ (18). Recent extensive analysis of HDV sequences has indicated that the various HDV genotypes fall into at least seven clades (19) and in a study (20) proposed an extended classification of the delta virus genus to eight clades. Most studies suggest that most HDV infections are acquired through parenteral and sexual routes (21, 22). Thus patients undergoing hemodialysis and patients infected with HIV are at risk of acquiring HDV. Coinfection with HBV/HDV has been shown to increase the risk of complications and virologic failure in patients with HIV infection (23, 24). Clinical studies regarding the frequency and genotypes of HDV infection in high risk patients were limited in Iran.

## 2. Objectives

This study aimed to determine the frequency and genotypes of HDV among patients with HIV and those undergoing hemodialysis in Tehran, Iran.

## 3. Patients and Methods

In this study 720 individuals including 120 patients undergoing hemodialysis and 600 patients with HIV infection as high risk group were enrolled from February to June 2011. The samples were collected from the main dialysis center of Tehran and Iranian Research center for HIV/AIDS in Tehran. This project was approved by Pasteur Institute of Iran ethics committee, and informed consent was obtained from patients prior to their enrollment. All samples were tested for hepatitis B surface antigen (HBsAg), hepatitis C antibody (anti-HCV) and anti-HDV by enzyme-linked immunosorbent assay (ELISA). The commercial enzyme immunoassay kits used were as follows: HBsAg (Hepanosticka Biomerieux, Boxtel, The Netherlands), anti-HCV (Biorad, Segrate, Italy), and anti-HDV (Dia.Pro Diagnostic Bioprobes sri, Milano, Italy). Recombinant immunoblot assay (RIBA Innogenetics, Ghent, Belgium) was employed to confirm anti-HCV reactivity. Human immunodeficiency virus antibody (anti-HIV) was determined by ELISA (MP Biomedicals, Illkirch, France); with positive tests confirmed by Western blot assay (Diaplus, San Francisco, USA). CD4^+^ count was determined by flowcytometry and defined as cells/mm3. HDV-RNA was extracted from 200 ml of serum using High Pure Viral Nucleic Acid kit (Roche Diagnostics GmbH, Mannheim, Germany) following the manufacturer’s instructions. Then RNA was converted to cDNA, subsequently, cDNA was amplified, using a denaturation step at 95ºC for 3 min, followed by 40 cycles of denaturation at 95ºC for 45 sec, annealing for 45 sec at 61ºC, and extension at 72ºC for 50 sec. RT-PCR products were amplified further using nested PCR (95ºC for 3 min, followed by 35 cycles of 95ºC for 45 sec, 60ºC for 45 sec, and 72ºC for 45 sec) using Taq polymerase (Roche Diagnostics GmbH). The primers used in the RT-PCR/nested PCR were as follows:

E1 (outer; sense), CCA GGT CGG ACC GCG AGG AGG (858–881 bases)

E2 (outer; anti sense), ACA AGG AGA GGC AGG ATC ACC GAC (1312–1289 bases)

E3 (inner; sense), GAT GCC ATG CCG ACC CGA AGA G (883–906 bases)

E4 (inner; anti sense), GAA GGA AGG CCC TCA AGA ACA AGA (1288–1265 bases)

**Figure 1.**
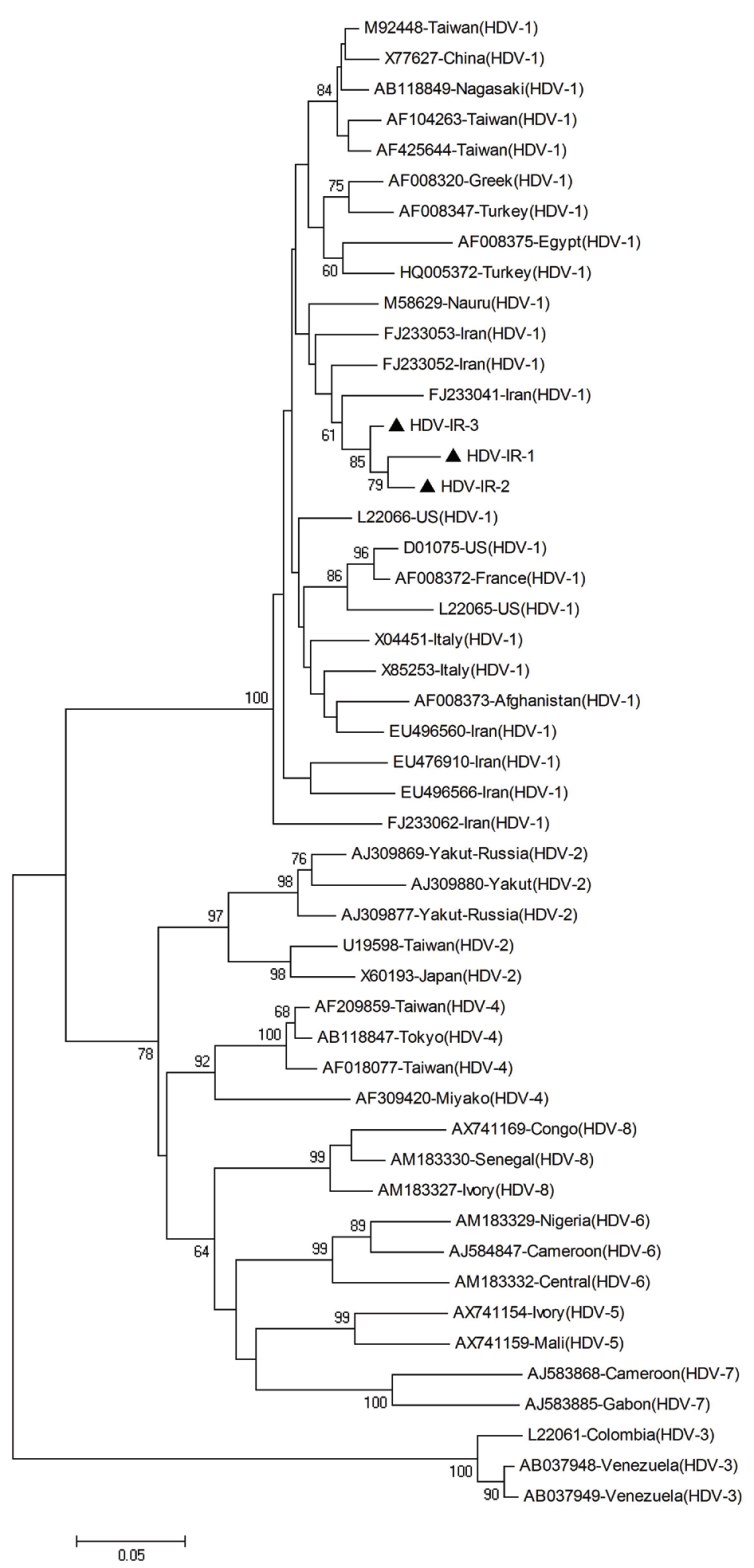
Phylogenetic Tree Constructed Using a Kimura Two-Parameter Matrix and the Neighbor-Joining Method of HDV Strains Iranian sequences determined in this study are indicated by the

The inner primers amplify a 404 base pair (bp) region in the C-terminal portion of the hepatitis delta antigen (HDAg) coding region and included the RNA editing site and the polyadenylation signal. PCR products were electrophoresed on a 1.5% agarose gel, containing ethidium bromide, and visualized on a gel documentation system. PCR products were purified using the High Pure PCR product purification kit (Roche Diagnostics GmbH, Mannheim, Germany), and sequenced directly at the Sequence Laboratories Gottingen GmbH (SEQLAB), Germany, using the inner primers. Sequences were compared with a set of published sequences corresponding to all the HDV clades shown in Figure 1.

Phylogenetic and molecular evolutionary analyses were conducted using MEGA version 4 (25) for phylogeny and BioEdit (The BioEdit Sequence Alignment Editor software, Department of the Microbiology, North California State University) for sequencing analysis. Nucleotide sequences were aligned with CLUSTALW program and confirmed by visual inspection. The Kimura two-parameter algorithm was used for genetic distance calculation (26). A phylogenetic tree was generated by the neighbor-joining method, and bootstrap resampling and reconstruction was performed 1000 × to confirm the reliability of the phylogenetic tree (27).

The HDV sequences used in this study were deposited in GenBank under accession numbers JF694492 through JF694494.

### 3.1. Statistical Analysis

The Chi-square was used with the SPSS 16 Package program for statistical analysis (Chicago, IL, USA). Data were presented as mean ± SD or, when indicated, as an absolute number and percentage.

## 4. Results

In this study 720 individuals including 120 patients undergoing hemodialysis and 600 patients with HIV infection were enrolled. The mean age of patients with HIV was 36.9 ± 9.2 (range: 9-67) years. 69.6% of patients were male and 30.4% were female. The mean CD4 ^+ ^count of patients was 275.6 ± 181 (16-1000) cells/mm3. The presumed routes of HIV transmission were intravenous drug use (53.6%), heterosexual contact (28.7%), infected blood and blood products transfusion (2.8%), vertical transmission (1.1%), tattooing (0.6%), injection drug use and tattooing (0.6%), heterosexual contact and intravenous drug use (4.4%), heterosexual contact and infected blood (0.6%), and in 7.6% the route of HIV acquisition was not identified. The mean age of patients undergoing hemodialysis was 55 ± 16 (range: 15-89) years. 60% of patients were male and 40% were female. The duration of hemodialysis was 5.2 ± 5.1 years. All of them had past history of blood transfusion, but none of them had organ transplantation. HBsAg was found in 9 (7.5%) of 120 patients undergoing hemodialysis and 9 (1.5%) of 600 patients with HIV infection. 3 (33.3%) of patients undergoing hemodialysis with positive results for HBsAg and 5 (55.5%) of cases with HIV infection and positive results for HBsAg, had positive findings for anti-HDV which were then subjected to nested PCR. The amplification results confirmed that in 3 (37.5%) samples HDV-RNA was detected. Two of them were under hemodialysis and one was HIV case. All HDV infected cases were male. All of the subjects coinfected with HIV/HBV/HDV (serology positive) were intravenous drug users, and were coinfected with HCV. Overall 2.5% of patients undergoing hemodialysis, and 0.8% of patients infected with HIV had positive results for anti-HDV, and 1.7% and 0.2% of cases undergoing hemodialysis and patients infected with HIV had positive findings for HDV-RNA respectively. Frequency of HBV and HDV infection in cases undergoing hemodialysis, and patients infected with HIV was summarized in Table 1.

**Table 1.** Prevalence of HBV and HDV Infection inPatients Undergoing Hemodialysis and Those with HIV Infection

Cases Characteristic	Patients undergoing Hemodialysis (n=120)	Cases Characteristic (n=600)
**Sex, Male/Female**	72/48	418/182
**Age, mean ± SD, y**	55±16	36.9±9.2
**HBsAg(+)^a^, No. (%)**	9 (7.5%)	9 (1.5%)
**Anti-HDV(+), No. (%)**	3 (2.5%)	5 (0.83%)
**HDV-RNA(+), No. (%)**	2 (1.66%)	1 (0.16%)

^a^Abbreviations: anti-HDV, hepatitis D antibody; HBsAg, hepatitis B surface antigen; HDV, hepatitis D virus

Phylogenetic analysis revealed that all HDV isolates were classified as clade 1 (genotype I) (Figure. 1).

## 5. Discussion

The prevalence of HDV is variable, predominating in some areas of the world (28). Most studies suggest that most HDV infections are acquired through parenteral and sexual routes (21, 22), thus cases undergoing hemodialysis and patients infected with HIV are at risk of acquiring HDV. The prevalence of HDV infection in patients infected with HIV and HBV ranges from 1.2% in the southeast region of Brazil to 14.5% in south and/or east Europe, and 22.2% in Taiwan (29-31). Coinfection is especially common among patients who are injection drug users (23, 24). Fainboim et al. (23) showed a low prevalence of anti-HDV in patients infected with HIV (1.9%). Anti-HDV was significantly higher in injection drug users (3.4%) than heterosexuals (0%). There was no significant difference in the percentage of anti-HDV positivity between injection drug users and homosexuals (1.0%). In another investigation, 3.9% of cases had triple infection with HIV/HBV/HDV (32). In a recent survey in Taiwan, 22.2% of patients infected with HIV with chronic HBV coinfection had positive findings for anti-HDV (30). A recently published study in Iran has shown a high HDV prevalence of 31.57% in individuals with HIV/HBV coinfection in Kermanshah, Western Iran (33). The current study showed that 1.5% of cases with HIV infection had HBV infection. It can be due to increased knowledge about HBV transmission routes, national vaccination program, and HBV vaccination of high risk patients. 0.8% of patients infected with HIV are coinfected with both HBV and HDV. All of the subjects coinfected with HIV/HBV/HDV were intravenous drug users. On the other hand, higher frequency of HDV infection (55.5%) was found among patients with HBV/HIV coinfection than that in the general population of Iranian HBsAg carriers (2.4-5.8%). The prevalence of HDV infection in patients with chronic hemodialysis is not clear (34). In studies by Ramia (35) and Voiculescu et al. (36), anti-HDV was not found in patients undergoing hemodialysis. Abraham et al. (37) reported that 2.9% of renal transplant recipients were infected with HDV. In an investigation in Iran, 44.5% of patients undergoing hemodialysis with HBV infection were infected with HDV (14). In another study in this country, 25.2% of patients undergoing hemodialysis and positive results for HBsAg were found to be anti-HDV positive (13). The findings showed that overall 33.3% of patients undergoing hemodialysis in Tehran had positive findings for anti-HDV which is similar to other reports from Iran. The results reveal relatively high frequency of HDV infection in cases coinfected with HIV/HBV (55.5%) and patients undergoing hemodialysis with positive results for HBsAg (33.3%), although overall HDV frequency might not be high in high risk groups. Recently, eight major clades of HDV (HDV-1 to HDV-8) were described. HDV-1 and HDV-3 are distributed worldwide, but other genotypes appear to be more restricted geographically. HDV-2 is found in Japan, Taiwan, and Yakutia, Russia; HDV-4 in Taiwan and Japan and HDV-5, -6, -7, -8 in Africa (20). The study revealed HDV clade I (genotype 1) in all of the patients. This result is in agreement with predominant genotype of HDV reported in other studies from Iran (18, 38), and also the Middle East region such as Lebanon Egypt, Turkey and Pakistan (17, 35, 39, 40). Previous findings indicated that there may be an association between the geographical distribution of HDV and HBV genotypes (38). As all reports from Iran showing that genotype D is the only detectable genotype in all clinical forms of HBV infection in this country (41, 42). It is expected that HDV genotype I was detected as predominant genotype in this study. In conclusion, this survey showed, although overall HDV frequency might not be high in the cases, an increase in HDV occurrence in the cohort of high risk patients were seen. Therefore, practitioners and health care managers should become aware of the risk of dual infection with HBV and HDV especially in high risk patients.
